# Supplementary material for: Two-Step Targeted Drug Delivery via Proteinaceous Barnase-Barstar Interface and Doxorubicin-Loaded Nano-PLGA Outperforms One-Step Strategy for Targeted Delivery to HER2-Overexpressing Cells
Source: Pharmaceutics. 2022 Dec 24;15(1):52. doi: 10.3390/pharmaceutics15010052 (PMC9861000; doi:10.3390/pharmaceutics15010052)
Supplement: Supplementary file 1 [file pharmaceutics-15-00052-s001.zip › pharmaceutics-2082486-supplementary.pdf]

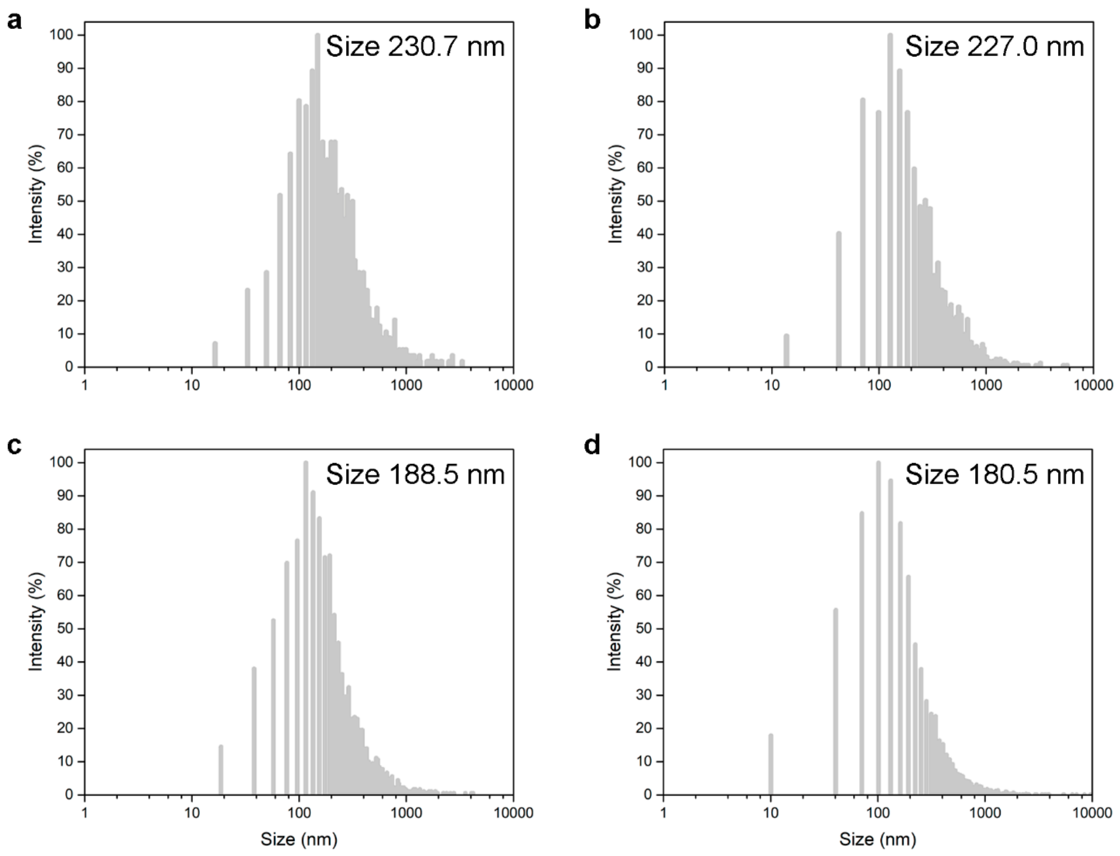

Figure S1. Physico-chemical properties of PLGA nanoparticles. Particle size distribution obtained with an AstraTrace (Abisense, Russia) for nanoparticles synthesized with 5 g/L (a), 1.7 g/L (b), 0.5 g/L (c), and 0.17 g/L (d) Nile Blue.

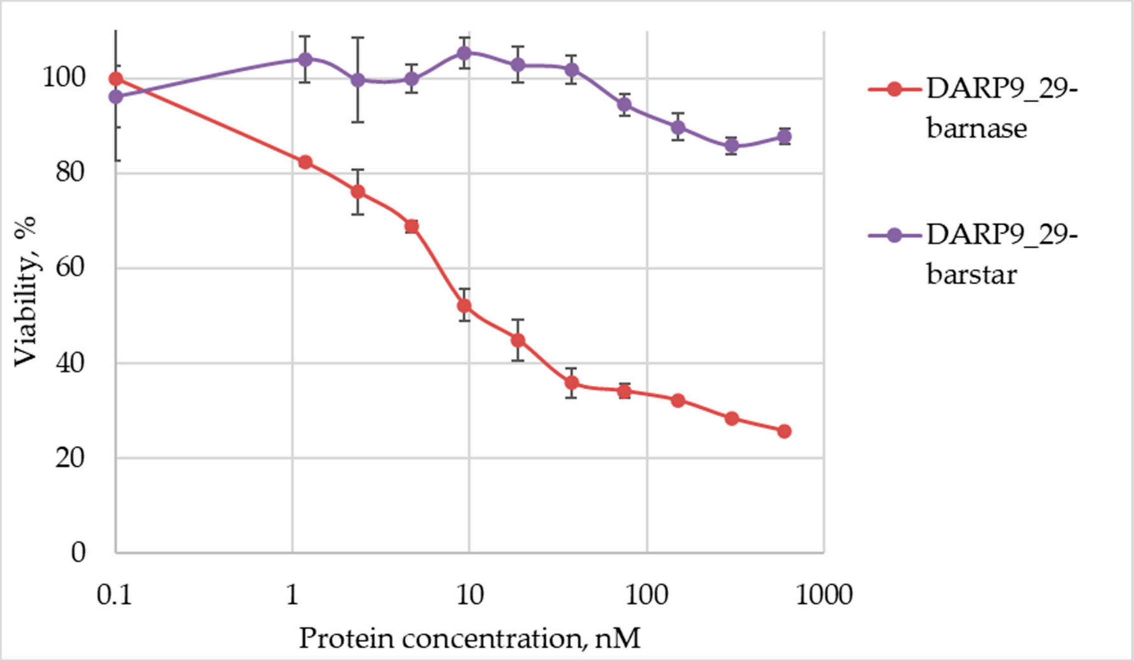

Figure S2. Cytotoxicity of DARP9_29-barnase and DARP9_29-barstar. BT-474 cells were incubated with proteins at different concentration and after 7 days of cultivation the MTT-test reflecting the number of viable cells was performed.
